# Comparative Dynamics of Retrograde Actin Flow and Focal Adhesions: Formation of Nascent Adhesions Triggers Transition from Fast to Slow Flow

**DOI:** 10.1371/journal.pone.0003234

**Published:** 2008-09-18

**Authors:** Antonina Y. Alexandrova, Katya Arnold, Sébastien Schaub, Jury M. Vasiliev, Jean-Jacques Meister, Alexander D. Bershadsky, Alexander B. Verkhovsky

**Affiliations:** 1 Ecole Polytechnique Fédérale de Lausanne, Laboratory of Cell Biophysics, Lausanne, Switzerland; 2 Belozersky Institute of Physical and Chemical Biology, Moscow State University, Moscow, Russia; 3 Department of Molecular Cell Biology, The Weizmann Institute of Science, Rehovot, Israel; 4 Cancer Research Center, Russian Academy of Medical Sciences, Moscow, Russia; 5 Institut “Biologie du Développement et Cancer” UMR 6543–CNRS, Nice, France; Max Planck Institute of Molecular Cell Biology and Genetics, Germany

## Abstract

Dynamic actin network at the leading edge of the cell is linked to the extracellular matrix through focal adhesions (FAs), and at the same time it undergoes retrograde flow with different dynamics in two distinct zones: the lamellipodium (peripheral zone of fast flow), and the lamellum (zone of slow flow located between the lamellipodium and the cell body). Cell migration involves expansion of both the lamellipodium and the lamellum, as well as formation of new FAs, but it is largely unknown how the position of the boundary between the two flow zones is defined, and how FAs and actin flow mutually influence each other. We investigated dynamic relationship between focal adhesions and the boundary between the two flow zones in spreading cells. Nascent FAs first appeared in the lamellipodium. Within seconds after the formation of new FAs, the rate of actin flow decreased locally, and the lamellipodium/lamellum boundary advanced towards the new FAs. Blocking fast actin flow with cytochalasin D resulted in rapid dissolution of nascent FAs. In the absence of FAs (spreading on poly-L-lysine-coated surfaces) retrograde flow was uniform and the velocity transition was not observed. We conclude that formation of FAs depends on actin dynamics, and in its turn, affects the dynamics of actin flow by triggering transition from fast to slow flow. Extension of the cell edge thus proceeds through a cycle of lamellipodium protrusion, formation of new FAs, advance of the lamellum, and protrusion of the lamellipodium from the new base.

## Introduction

Cell migration involves coordination of protrusion at the cell front, adhesion of the newly protruded domains to the substrate, pulling of the bulk of the cell towards new adhesion sites, and breaking of adhesion and retraction at the cell rear. These events are largely based on actin microfilament system: protrusion is believed to be driven by assembly of actin network at the leading edge, while generation of the tension force to pull the cell body and retract the tail depends, at least in part, on the contraction of actin network by motor protein myosin II [1], [2]. Adhesion of actin network through plasma membrane to the extracellular matrix is mediated by specialized protein complexes termed focal adhesions (FAs) [3]–[5], which also serve as signal transduction sites where the cell gathers information about the mechanical and chemical properties of the environment [3], [6].

The interaction between FAs and actin network is complex, in particular due to the fact that actin network in most migrating cells is not stationary with respect to the substrate, but moves away from the leading edge of the cell in a process known as retrograde flow [7], [8]. Retrograde flow is thought to be a consequence of the same forces that drive cell migration: the pressure of actin assembly against the membrane and contractile forces in the actin network [9], [10]. FAs in some instances have been observed to move themselves [11]–[13], but the majority of FAs at the leading edge of the cell is stationary [14]. To rationalize the relationship between adhesion and retrograde flow, a hypothesis has been put forward likening adhesion to a clutch [9], [11], [15], [16]. When the clutch is not engaged (no adhesion), actin machinery runs idle resulting in retrograde flow but no net advance of the cell; conversely, establishment of adhesion converts actin network flow into a productive advancement of the cell. Consistent with this hypothesis, inverse correlation between cell advance and retrograde flow rate was indeed demonstrated in some cases [11], [17], but not in other cases [17], [18]. In most cases, retrograde flow coexists with adhesion, suggesting that FA components form a slipping interface with the actin network. Recent studies demonstrated various degrees of correlation between motion of filamentous actin and various adhesion components, establishing a hierarchical order of interaction within this interface [19], [20]. However, a more general question of how FAs arise in the midst of flowing actin network and if and how their formation influences the flow remains unanswered.

The relation between actin network and FAs is further complicated by the existence of two different types of actin network at the leading edge of the cell, each having its characteristic composition, dynamics and flow velocity [21], [22]. The more peripheral network domain, the lamellipodium, is characterized by faster actin turnover and retrograde flow than the inner domain, the lamellum. Signature components of the lamellipodium include Arp2/3 and ADF/cofilin, involved in formation and turnover of branching actin network, while the lamellum contains tropomyosin and myosin II, proteins characteristic of contractile bundles of actin filaments. Mature FAs which are associated with contractile actin bundles, are also abundant in the lamellum [19], [22].

The relative roles of the lamellipodium and the lamellum in the cell are controversial. One point of view is that lamellipodium is the “organ of protrusion” and the factory of actin filaments in the cell [23]. Other studies suggest that most of the actin filaments assembled in the lamellipodium disassemble locally and do not become incorporated into other actin structures in the cell, and that productive advance of the cell correlates with the advance of the lamellum rather than the lamellipodium [22], [24]. However, the mechanism of the advance of the lamellum is not clear. While the lamellipodium displays a zone of intense actin assembly at its outer edge, consistent with the lamellipodial protrusion being powered by actin assembly, the lamellum is characterized by a distributed pattern of actin turnover [22]. Consequently, it is not clear if the expansion of the lamellum proceeds via local assembly of the lamellar actin network at its outer edge, or via acquisition of actin filaments from the lamellipodium. Importance of the lamellipodium actin assembly emerged from the analysis of the origin of actin filament bundles in the lamellum [25]. This study suggested that transverse actin arcs formed in Arp2/3-depended mechanism characteristic of the lamellipodium, while dorsal stress fibers arised in the arp2/3-independent, formin-dependent manner. Thus, one population of actin filaments in the lamellum may be inherited from the lamellipodium, while another population may arise locally. Recent analysis of the dynamics of cell spreading [26], [27] suggested that edge extension is a cyclical process involving expansion and retraction of the lamellipodium, and that the lamellipodial actin provides a transient link between myosin II and adhesion sites. Irrespective of the eventual fate of the lamellipodial actin filaments, it is unclear how the position of the lamellipodium/lamellum boundary is defined and why this boundary moves during cell spreading and migration.

In this report, we analyze the correlative dynamics of FAs and the boundary between the lamellum and the lamellipodium in spreading cells. We find that formation of FAs depends on actin flow and at the same time controls the dynamics of flow the by triggering the transition from fast to slow flow and defining the position of the lamellipodium/lamellum boundary.

## Results

### Two types of retrograde flow visualized by enhanced phase contrast microscopy at the periphery of spreading cells

**Figure 1:**
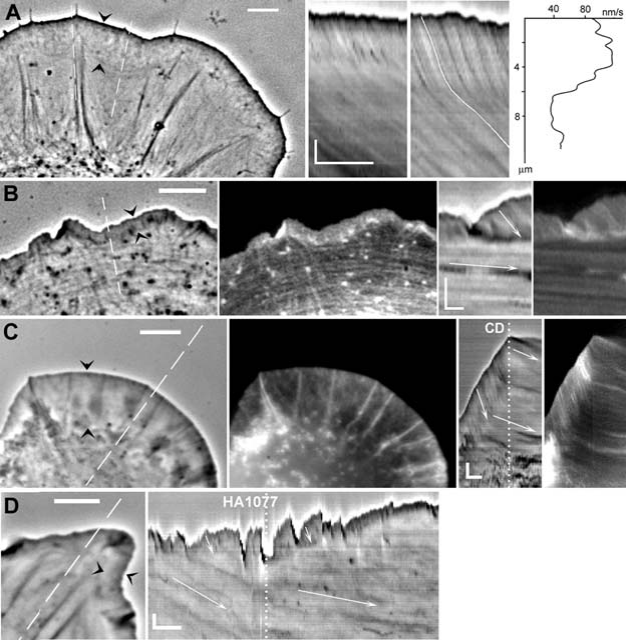
Retrograde actin flow in spreading cells visualized by enhanced phase contrast and fluorescence microscopy. Individual images of the time-lapse sequences are shown at the left, kymographs, at the right. Arrowheads indicate zones of fast flow (lamellipodia) , dashed lines on images indicate the regions used to generate kymographs, arrows in kymographs indicate the slope of the isointensity lines reflecting the velocity of flow. (A) Swiss 3T3 fibroblast at 1.5 h of spreading displays wide lamellipodium, which is distinguishable from the lamellum by its density and texture (left image) and the velocity of retrograde flow (see Supplementary Data, Movie S1A). Kymographs are generated along the phase-dense fiber (second from the left) and along the line between the fibers (second from right). The isointensity line in kymograph was traced manually (white line) and analyzed in Matlab to generate the plot of velocity versus distance from the edge of the cell (right), which shows an abrupt change of velocity from approximately 6.2 µm/min to 2.4 µm/min at the lamellipodium/lamellum boundary. (B) REF-52 fibroblast at 5 h of spreading injected with rhodamine-actin and visualized with double mode microscopy: phase contrast (left) and fluorescence (second from left). The lamellipodium is distiguishable from the lamellum by its high actin concentration and the density and texture in the phase contrast images. The width of the lamellipodium in late spreading was smaller than in recently plated cells (compare with A). Small fibers in the lamellum are apparent in both fluorescence and phase contrast images. Kymographs generated from the phase contrast (second from right) and fluorescence (right) image sequences show identical flow velocities (0.8 µm/min in the lamellipodium, and 0.06 µm/min in the lamellum). See also Supplementary Data, Movie S1B. (C) Spreading B16 melanoma cell expressing GFP-actin imaged in double phase contast/fluorescence mode and analyzed with kymographs as in (B). Dashed line marked “CD” on kymograph indicates the time of addition of cytochalasin D (2 µM). Addition of cytochalasin D resulted in the immediate arrest of spreading and the decrease of the velocity of the lamellipodial flow (from 2.6 µm/min to 0.4 µm/min), which thus became equal to the velocity of the lamellar flow. (D) Enhanced phase contrast image (left) and kymograph (right) of spreading Swiss 3T3 cell. Dashed line on kymograph indicate the time of addition of 30 µM HA1077, which results in the decrease of velocity of the lamellar flow (from 1 to 0.4 µm/min) with no change in the lamellipodial flow (4.5 µm/min). Scale bars on images, 5 µm; on kymographs, vertical bars, 2 µm, horizontal bars, 2 min.

Enhanced phase contrast microscopy [28] allows detection of small contrast differences in the peripheral cytoplasm. It was previously used to analyze the organization [28] and dynamics [29] of the actin network in the lamellipodium of migrating fish keratocytes. Here, we applied this technique to visualize retrograde flow and the lamellipodium/lamellum transition in spreading fibroblasts and melanoma cells (Fig. 1). Comparison of enhanced phase contrast images and fluorescence actin images demonstrated that all the actin structures (lamellipodial network, filopodial-type small filament bundles, and stress fibers terminated at adhesion plaques) that were visualized in the fluorescence images were also clearly detectable in the enhanced phase contast images (Figure 1B, C). We followed the dynamics of these features in time-lapse movie sequences of the phase contrast images and fluorescence images. Two distinct zones of retrograde flow from the cell edge to its center were clearly distinguishable in time-lapse sequences and resolved by kymograph analysis (Figure 1 and Supporting Movies S1 and S2). The peripheral (faster) zone of flow coincided with the lamellipodial network of actin (Figure 1, arrowheads), while the more central (slower) zone encompassed the region of the cytoplasm containing stress fibers and commonly defined as the lamellum. Flow velocity varied depending on the type and state of the cell (0,5–7 µm/min for the lamellipodium and 0,05–2,5 µm/min for the lamellum, with faster flow typically observed at the early stages of spreading), but in the same cell flow velocity in the lamellipodium was always higher than that in the lamellum. Interestingly, the flow of the phase contrast features along the radial stress fibers in the lamellum and in the space between the stress fibers proceeded with exactly the same velocity, while the positions of the peripheral ends of the stress fibers did not change with time (Figure 1A). The lamellum and the lamellipodium were previously distinguished by their kinematics and dynamics using fluorescence speckle microscopy [21], [22].

Consistent with the previous reports [10], [22], our phase contrast microscopy analysis revealed that two types of retrograde flow had different inhibitor sensitivities and therefore were likely driven by different mechanisms. The lamellipodial flow was instantly inhibited upon treatment with cytochalasin D (Figure 1C, and Supporting Movie S3). At the same time, up to two-minute treatment with cytochalasin D did not affect the velocity of flow in the lamellum. Inhibition of cell contractility with the rho-kinase inhibitor HA1077 [30] (Figure 1D and Supporting Movie S4) and the protein kinase inhibitor H7 [31] (not shown), on the other hand, reduced the velocity of lamellar flow and did not affect lamellipodial flow.

Kymograph analysis indicated that the flow velocity changed abruptly at the boundary between the two zones of flow (Figure 1A). This boundary was distinct based on both velocity analysis and the change of density and texture of the actin network seen in fluorescence as well as in phase contrast images (Figure 1A–D). Interestingly, the boundary was not smooth, but often consisted of several segments, convex in the inside direction, forming a festooned line. The apexes of this line coincided with the termini of the radially oriented stress fibers, which likely corresponded to the FAs (Figure 1A). Thus, it appeared that the position of the boundary between the two flow zones was related to the positions of FAs. Next, we analyzed the relationship between flow and adhesion sites in more detail.

### Formation of nascent adhesions locally blocks the lamellipodial flow

**Figure 2:**
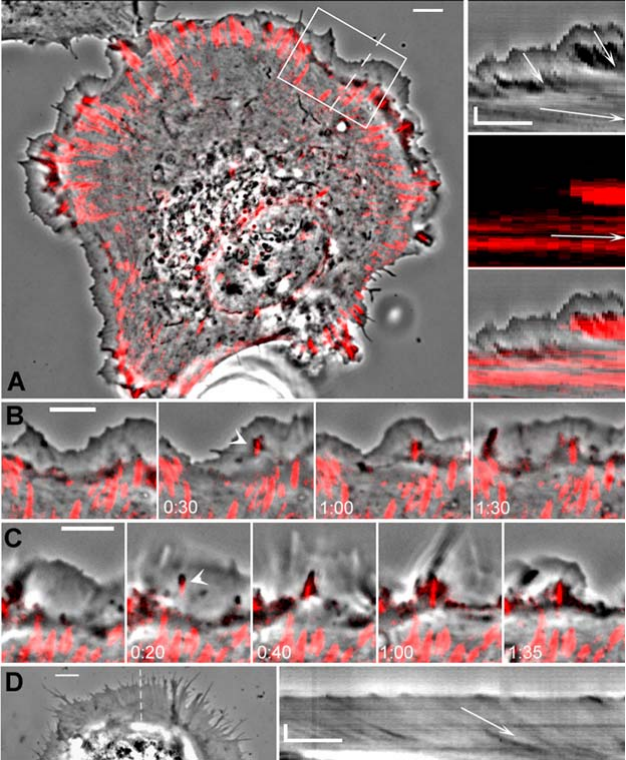
FAs define the boundary between fast and slow flow. (A) Superimposition of the enhanced phase contrast and fluorescence images (left) and kymographs (right) of spreading REF-52 cell expressing YFP-paxillin. Phase contrast image is represented in gray scale, and YFP-paxillin fluorescence image, in red. FAs marked by YFP-paxillin coincide with the boundary between the lamellipodium and the lamellum (see also Supplementary Data, Movie S2A). Kymographs (phase contrast on top, fluorescence in the middle, and merge at the bottom) demonstrate that the fast flow did not penetrate behind the FAs and that the zone of slow flow advanced concomitantly with the formation of new FA. Arrows on kymographs are drawn parallel to isointensity lines indicating the velocity of fast flow (4.5 µm/min), slow flow (0.5 µm/min), and the velocity of the sliding of FAs (0.15 µm/min). (B, C) Selected frames from the time-lapse sequence showing two instances of the formation of the new FAs and associated dynamics of the boundary between the two flow zones; time is indicated in minutes:seconds. Sequence (B) represents dynamics of the region boxed in (A). New FAs (paxillin-positive spots indicated with arrowheads) form within the lamellipodia at 30 s in (B), and 20 s in (C). Disturbance of the flow is simultaneously seen in the phase contrast image as a dark zone in front of the FA. Formation of FAs is followed next by the advance of the lamellum (dark boundary between the lamellipodium and the lamellum is visible in the phase contrast mode). In (B) the lamellipodium persists throughout the sequence, while in (C) formation of the new FA is followed by the ruffling and withdrawal and then re-growth of the lamellipodium. See Supplementary Data, Movies S2B and S2C. (D) REF-52 cells were plated in the serum-free media onto the coverslips coated with poly-L-lysine (1 h with 10 mg/ml aqueous solution), kymograph generated along the dashed line at the left is shown at the right. Kymograph demonstrates uniform flow velocity (1.6 µm/min) throughout the spread part of the cell. See also Supplementary Data, Movie S2D. Scale bars, 5 µm; in kymographs vertical bars, 2 µm, horizontal bars, 2 min.

We investigated simultaneous dynamics of FAs and retrograde flow using YFP-paxillin as a marker of adhesion sites. Time-lapse sequences recorded in double enhanced phase contrast /fluorescence mode demonstrated that the boundary between the fast and slow flow zones coincided with the line connecting the outmost FAs at the cell periphery (Figure 2A and Supporting Movie S5). During cell spreading, new paxillin-positive sites (nascent FAs) always appeared outside of the area bordered by this line (194 events of new adhesion formation observed in 8 spreading cells), indicating that they originated within the lamellipodia. In the double-mode image sequences, nascent FAs were indeed initially detected within the lamellipodial flow zone. Within seconds of the detection of the nascent adhesion, phase contrast microscopy revealed irregularity of rapid lamellipodial flow manifesting as an accumulation of phase dense material at the site of adhesion and an abrupt reduction of the flow velocity in the zone immediately behind the adhesion site (Figure 2B, C and Suppurting Movies S6 and S7). This disturbance of flow may be the cause for previously noted phenomenon of “ruffling” at the new adhesion sites [32]. As the new adhesion increased in size, the new boundary between the lamellipodial and the lamellar flow zones appeared, passing through the site of the newly formed adhesion (Figure 2B, C). The multiple events of the formation of new adhesions in the lamellipodial flow zone eventually resulted in the gradual anterograde movement of the boundary between the lamellum and the lamellipodium. Advance of the boundary was often associated with a transient decrease in the width of the lamellipodial zone, but the lamellipodium subsequently re-grew to its original width due to the protrusion at its outer boundary (Figure 2C, and Supporting Movie S7). Thus, anterograde movement of the boundary between the flow zones in combination with the lamellipodial protrusion contributed to the overall spreading of the cell. The coupling between the formation of new adhesions and the advance of the lamellipodium/lamellum boundary was evident both in the enhanced phase contrast/YFP-paxillin double imaging mode and in the rhodamine-actin/YFP-paxillin double fluorescence mode (Supporting Movie S8).

The advance of the boundary between fast and slow flow did not always coincide with the maturation of small dot-like adhesions into large streak-like structures. In some cases, nascent adhesions did not mature into large adhesions, but still marked the boundary between the fast and slow flow. Advance of the boundary was associated with apparent rapid turnover of these adhesions (Supporting Movie S9).

**Figure 3:**
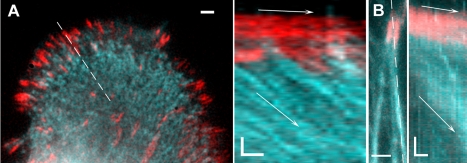
Flow of the components of stress-fibers with respect to FAs. (A) Double fluorescence image (left) and kymograph (right) of YFP-paxillin (red) and rhodamine-myosin II (cyan) in REF-52 cell. Myosin flow velocity is 1 µm/min, FAs move at 0.05 µm/min (arrows). See Supplementary Data, Movie S3A. (B) Double fluorescent image (left) and kymograph (right) of YFP-paxillin (red) and GFP-actin (cyan) in REF-52 cell. Actin flow velocity in the stress fiber is 0.75 µm/min, FAs move at 0,12 µm/min (arrows). See Supplementary Data, Movie S3B. Scale bars, 2 µm; in kymographs vertical bars, 2 µm, horizontal bars, 2 min.

Slow retrograde flow persisted behind the adhesion zone and, as noted above, was observed by phase contrast microscopy not only within the bulk of the lamellum, but also along the phase-dense cables likely representing the stress-fibers terminating in focal adhesion sites. Consistent with recent reports [25], [33], this was confirmed by double fluorescence imaging of the markers of stress fibers and focal adhesions. We used myosin II as one of the markers of stress fiber dynamics, because it was previously shown that myosin II is distributed in the lamellum and along the stress fibers in the form of distinct clusters of bipolar minifilaments [34]. Double florescence imaging with microinjected rhodamine-myosin II and YFP-paxillin demonstrated a flow of myosin-positive spots to the center of the cell away from the adhesion sites with velocities similar to the velocity of the lamellar flow component (Figure 3A, and Supporting Movie S10). Focal adhesions remained stationary or moved at velocities much smaller than that of the myosin flow. This flow depleted myosin from the area immediately behind the adhesion, but new myosin spots continuously appeared there, so that the overall myosin density remained virtually constant. The flow along the fibers with respect to their associated adhesion sites was also confirmed using fluorescence imaging of the cells either injected with fluorescent actin probe or expressing GFP-actin probe. Distinct specific features along the fiber (e.g. bifurcations) and non-uniformity of actin density served as reference points (Figure 3B and Supporting Movie S11).

The above findings suggested that FAs defined the position of the boundary between two flow zones. To analyze the organization of the flow in the absence of FAs, we plated the cells on poly-L-lysine-coated surface in the absence of serum and extracellular matrix proteins. In these conditions, the cells attach and spread, but the specific FA complexes and stress fibers do not develop [35]. The cells initially spread rapidly on poly-L-lysine, but subsequently arrested in a “fried-egg” state with the bulk of the cytoplasm remaining in the center. The spreading domain appeared uniform in its organization and dynamics from the edge to the perinuclear cytoplasm. It exhibited retrograde flow with the velocity either uniform or gradually decreasing towards the cell center and intermediate in value between typical fast and slow flow velocities (Figure 2D and Supporting Movie S12). Thus, two zones of flow did not develop in the absence of FAs, suggesting that the boundary between these zones not only coincided with, but indeed depended on FAs.

### Inhibition of the lamellipodial flow blocks the formation of nascent adhesions

**Figure 4:**
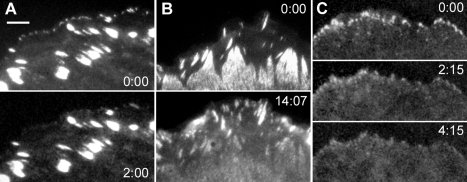
Effect of the inhibitors of retrograde flow on FAs. FAs were visualized with YFP-paxillin. FA distribution is shown just before (top panels in A–C), and after the addition of the inhibitor (bottom panels in A–C, and middle panel in C), time after the addition is indicated in minutes. (A) Cytochalasin D treatments (2 µM) abolished small nascent adhesions; (B) H7 treatment (30 µM) abolished large mature adhesions and increased the number of small adhesions; (C) cytochalasin D treatment of the cells preincubated for 1 h with H7 abolished small adhesions which were present after treatment with H7. Bar, 5 µm.

In the previous section, we have demonstrated that nascent adhesions formed exclusively in the fast flow zone. Next, we investigate if the experimental inhibition of fast flow affects the formation of nascent adhesions. We studied paxillin dynamics during a short-term treatment with cytochalasin D, which, as shown in the first section, rapidly abolished the fast flow. We did not observe formation of any new nascent adhesions at the cell periphery, moreover, the small peripheral adhesions present before addition of the drug disappeared after approximately two minutes of treatment (Figure 4A). At the same time, large mature adhesions remained unaffected.

In contrast to the inhibition of fast flow, inhibition of slow flow with the inhibitors of cell contractility increased the number of small dot-like adhesions (Fig. 4B). This can be explained by the inhibition of maturation of these small adhesions into large ones upon block of contractility as was previously reported in several studies [31], [36], [37]. Consistent with the results of the same studies, rho-kinase inhibition also led to disassembly of previously existing mature adhesions (Figure 4B). Finally, combined treatment with rho-kinase inhibitors and cytochalasin D resulted in total disappearance of both types of adhesions (Figure 4C).

## Discussion

In this study, we analyzed comparative dynamics of actin retrograde flow and focal adhesions at the leading edge of the cell. Many previous studies investigated actin dynamics at the leading edge and the dynamics of focal adhesions separately [10]–[14], [21], [22], [38]. A few recent studies also analyzed simultaneous dynamics of actin and the FA components [19], [20], but did so in a steady state situation where the formation of new FAs was not observed. In contrast to these studies, we concentrated on the changes of actin flow associated with the formation of new FAs during cell spreading and on the interaction between actin dynamics and adhesion dynamics.

Two zones of retrograde flow at the cell periphery were previously observed with fluorescent speckle microscopy technique [21], and distinguished using automated analysis of speckle dynamics [22]. Here we visualized flow using enhanced phase contrast microscopy [28], [29]. The advantages of this technique are that no exogenous markers for actin network need to be introduced into the cell, and that the flow is analyzed in the phase contrast images that simultaneously show overall cell morphology. Enhanced phase contrast microscopy clearly visualized two types of the flow. The boundary between two flow zones was clearly distinguishable in our images both morphologically and with kymograph analysis, which demonstrated an abrupt change of the flow velocity typically by a factor of two or more at the boundary. The velocities of flow varied significantly, with the maximum values (7 µm/min for fast flow, and 2,5 µm/min for slow flow) being higher than previously reported (e.g., 0.5–2 µm/min for fast flow, and 0.1–0.3 µm/min for slow flow in [21], [22]), suggesting that flow velocities in both zones may depend on the type and/or physiological state of the cell. In particular, high flow velocities may be associated with the early state of radial cell spreading. Interestingly, velocities of the fast retrograde flow in spreading cells measured here are of the same order of magnitude as the migration velocities of rapidly migrating cells, e.g., fish epidermal keratocytes [17], [39]. This finding indicates that maximal actin assembly rates may be similar in different types of cells, while the difference in protrusion rate between the “slow” cells (fibroblasts) and “fast” cells (keratocytes) may be due to the difference in retrograde flow rate (high in spreading fibroblasts and low in migrating keratocytes).

We have correlated the patterns of retrograde flow with the dynamics of FAs in the same cells and found that 1) positions of FAs at the cell periphery coincided with the boundary between the fast and slow flow; 2) nascent adhesions first appeared in the fast flow zone; 3) the boundary between the fast and slow flow advanced to the new adhesion sites within seconds of the formation of new adhesions; and 4) in the absence of FAs (on polylysine-coated substrate) flow was apparently homogeneous and not separated in two zones. Thus, FAs not only appeared necessary for the formation of the lamellipodium/lamellum boundary, but their location seemed to define the position of the boundary. Since formation of new FAs was a transient event and was followed by rapid advance of the lamellum to the new FA sites, static observations demonstrated co-localization of the lamellipodium-lamellum boundary with the most peripheral row of Fas. This co-localization was also noted in a previous study [19], although the relation of the movement of the lamellum/lamellipodium boundary to the formation of new adhesions was not analyzed. Also consistent with our findings, sharp boundary between the two zones of flow was not observed in sea urchin coelomocytes cultured on polylysin-coated surfaces [10], , although inhibitor analysis revealed two different components of flow velocity. Taken together, these findings strongly suggest that the formation of FAs is the critical event controlling the transition from the fast to the slow flow type and that FAs define the position of the boundary between the lamellipodium and the lamellum.

How FAs influence the flow and what exactly are the features of FAs that trigger the formation of the boundary between the fast and slow flow is a question for future research. Our observations that the boundary advances within seconds of the formation of FAs and that small FAs that turn over at the edge of the cell and do not mature into larger long-living Fas (see Supporting Movie S9) are sufficient for the formation of the boundary suggest that maturation of FAs is not necessary for the effect on actin flow. The simplest hypothetical mechanism of how nascent adhesions modulate the flow is the mechanical resistance due to the anchoring of actin filaments to nascent adhesion complexes. Similar event may have been previously observed in an artificial situation: arrest of the flow upon anchorage to a synthetic bead on the apical cell surface [40]. One can imagine that at first a few filaments become anchored, than more filaments arrive with the flow and become entangled and anchored, and eventually the network flow is locally arrested. However, initial mechanical arrest of the flow may represent just a first step in the formation of the lamellipodiam/lamellum boundary and may be by itself not sufficient for the demarcation of the two types of actin network. Initial mechanical events may trigger a signaling cascade resulting in a change in the overall organization and composition of the actin network, which could me mediated by a change of activity of actin-interacting proteins, e.g. controlling actin filament stability such as cofilin and tropomyosin [41].

Another open question is how the discrete FAs create continuous boundary for the network flow. One possibility is that the continuous barrier forms due to the cross-linking and entanglement of the filament network between discrete pin-down points of individual FAs. In this mechanism, one could expect that the segments of the boundary between adjacent FAs would bend inward due to the exterior pressure from the fast flow, apparently consistent with the observed shape of the boundary (convex inward between FAs, see Fig. 1A). Quantitative analysis of the stress distribution pattern around FAs in the moving actin network is a challenge for biophysical modeling and may help to understand the nature of the lamellipodium/lamellum boundary.

Several recent studies [22], [27] suggested a partial overlap between the lamellum and the lamellipodium. In our experiments, the boundary between the two zones always appeared distinct, except immediately after the formation of new adhesion, when the boundary shifted its position. Further time-resolved analysis is necessary to determine whether the overlap between the two zones is limited to the phases of cell protrusion when the boundary is shifting, or exists continuously.

While formation of FAs apparently creates a barrier for the fast flow, actin network inside the area bordered by FAs is not completely immobilized, but undergoes slower flow characteristic of the lamellum and probably driven by myosin-dependent contraction. The flow of the components of stress fibers away from the stationary FAs associated with their tips suggests that stress fibers grow at these tips. The growth may represent either a de novo assembly of actin filaments, or an association with the fiber tip of the pre-assembled actin filaments delivered by flow. In any case, the growth mechanism should allow for continuous connection and force transmission between the growing stress fiber tip and focal adhesion complex. Recent study [25] suggested that formin-family proteins that form leaky cups at the barbed ends of actin filaments [42], [43] may be responsible for assembly of at least a part of the population of actin filaments in filament bundles in the lamellum.

**Figure 5:**
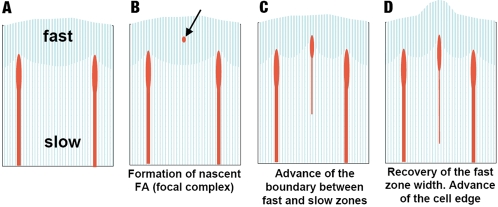
Diagram of the multi-step protrusion process at the leading edge of the cell. Fast and slow flow zones are shown with different shading; nascent and mature FAs are shown as ellipses of different sizes; and stress fibers are shown as sticks. Formation of nascent FAs within the fast flow zone locally interferes with flow, and eventually results in the advance of the boundary between the fast and slow flow zones. Therefore, the width of the slow flow zone (the lamellum) increases, while the width of the fast flow zone (the lamellipodium) decreases. Subsequently, the lamellipodium re-establishes its width, resulting in the overall advance of the cell edge.

Our results suggest that protrusion at the leading edge of the cell could be considered as a cycle of alternating steps of protrusion of the lamellipodium and advance of the lamellum, with the formation of FAs linking these steps together (Fig. 5). This cycle may be related to the recently described periodic lamellipodial contractions in spreading epithelial cells [26], [27]. However, unlike [27], we do not find a correlation between the retraction phase of the cycle and the formation of new FAs. In our experiments, new FAs appeared during lamellipodial advance as well as during its temporal retraction.

Ponti et al. [22] argued that the advance of the cell edge is correlated with the advance of the lamellum rather than the lamellipodium. These authors considered actin dynamics in the lamellum autonomous from the lamellipodium, with very little material transfer between the two. However, single speckle tracking techniques may tend to underestimate the transfer of polymer mass at the lamellipodium/lamellum transition where the speckle velocity changes abruptly and individual speckles may be difficult to follow. Part of the actin filaments of the lamellum may originate in the lamellipodium [25]. Irrespective of whether any actin filaments from the lamellipodium are transferred to the lamellum or not, our findings indicate important role of the lamellipodium in the protrusion cycle. The lamellipodium emerges as the site where nascent adhesions arise. Moreover, we found that the inhibition of the lamellipodial actin flow abolishes nascent adhesions, suggesting that flow is necessary for their formation and maintenance. What are the specific features of the actin dynamics in the lamellipodium that promote the initiation of adhesions? One of the possibilities is that intensive actin polymerization in the lamellipodium creates conditions where the adhesion formation is favored. DeMali et al. [43] suggested that Arp2/3 complex involved in branching actin assembly may recruit FA component vinculin and promote its association with actin. Alternatively, the flow itself may stimulate adhesions mechanically, e.g. promote clustering of the adhesion molecules or trigger hypothetical mechanical sensors activating the adhesion process. We find the last possibility especially intriguing, as it would suggest a peculiar symmetry between the initiation and maturation of the focal adhesions. It is well established that the maturation of focal adhesions is promoted by applied tension, suggesting a stretch-activated sensor mechanism [45]–[48]. We propose that nascent adhesions may also depend on force, but instead of myosin-dependent contraction force involved in maturation, nascent adhesions may depend on force associated with fast network flow in the lamellipodium. Displacement of adhesion components with respect to extracellular matrix was proposed as a key element of mechanosensing [49]. Analogously, relative movement of adhesion proteins and lamellipodial actin network may be involved in maintenance of nascent adhesions and their maturation. The specificity of the chemical and mechanical environment in the lamellum and the lamellipodium may define specific force- and/or displacement-triggered responses. Interestingly, “young” adhesions at the lamellipodium/lamellum boundary may experience both the compression due to the external “push” of the lamellipodial flow and stretching due the “pull” of the lamellar flow, possibly explaining strong traction at the substrate [50]. Irrespective of the specific molecular mechanisms of force-triggered responses that remain a challenge for the future, our study elucidates the essential reciprocal relation between dynamics of actin flow and FAs.

## Materials and Methods

### Cell culture

Swiss 3T3 mouse fibroblasts, REF-52 rat fibroblasts and B16 mouse melanoma cells were cultured in DMEM supplemented with 10% fetal bovine serum and antibiotics. For microscopy, the cells were plated at low density in 35 mm Petri dishes with heated glass bottom (Bioptechs, Butler, PA), or into the home-made Petri dishes with coverslip glued over the hole in the bottom. Before the observation, the culture medium was replaced with fresh DMEM with 25 mM HEPES, 10% fetal serum and antibiotics. 1–1.5 ml per dish of mineral oil was then applied to the surface to avoid evaporation on the microscope stage. The temperature was maintained during observation using the Bioptechs (Butler, PA) dish and objective heaters, or an infrared lamp.

### Cytoskeletal markers

Rhodamine-actin and rhodamine-myosin II were prepared and microinjected as described [34], [51]. To produce YFP-paxillin construct, paxillin cDNA (kindly provided by K. Nakata, S. Miyamoto and K. Matsumoto, National Institute of Dental and Craniofacial Research, NIH, Bethesda, MD) was cloned into EYFP-C1 (Clontech Laboratories, Palo Alto, CA, USA). GFP-actin construct [52] was kindly provided by G. Marriott (Max-Plank-Institute for Biochemistry, Martinsried, Germany). To achieve transient expression of YFP-paxillin and GFP-actin Swiss 3T3 and REF-52 cells were transfected with either one or both of the constructs by nuclear microinjection.

Stable GFP-actin expressing B16 melanoma cell line [53] was a generous gift of Christoph Ballestrem (Weizmann Institute of Science) and Bernhard Wehrle-Haller (University of Geneva). Stable YFP-paxillin expressing REF-52 cell line was produced using retroviral infection. To produce vector for retroviral infection, DNA encoding YFP-paxillin was cut from pEYFP-paxillin expression vector and ligated into a retroviral vector, pBabeNeo [54]. The vector was expressed in packaging cell line, as described [54], and supernatant containing viral particles was used to infect REF-52 cells. Following infection, cells were selected with G418 and were further cultivated in the G418-containing medium (1 mg/ml).

### Microscopy

Optical microscopy was performed using a Nikon Eclipse TE300 inverted microscope with CFI Plan 100x phase objective (NA 1.25) and 100 W halogen and 100 W HBO light sources for phase contrast and epifluorescence microscopy, respectively. Images were captured with a Roper Scientific (Tucson, AZ) MicroMAX-1300PB cooled CCD camera operated with Metamorph software (Universal Imaging, West Chester, PA). To switch automatically between phase contrast and multicolor epifluorescence microscopy modes, shutters in the transmitted and epifluorescence light paths and exitation and emission filter wheels were operated with Ludl Electronic Products Ltd. (Hawthorne, NY) MAC 2000 and MAC 5000 controllers driven with Metamorph software. XF52-1 Pinkel filter set (Omega Optical, Brattleboro, VT) was used to separate rhodamine and YFP fluorescence, and JP3 filter set (Chroma Technology Corp., Rockingham, VT) was used to separate GFP and YFP fluorescence.

Enhanced phase contrast microscopy was performed as described [28] (Verkhovsky et al., 2003): briefly, single frame exposures of 500–800 ms were used to achieve intensity readout of about 50% saturating level of the camera. Background images of the cell-free areas of the same dish were subtracted from the cell images (a constant was added to the result to avoid negative intensity values), and the resulting images were scaled to a narrow contrast range for optimal visualization of the structure of the lamellum and the lamellipodium.

Image acquisition rate was one frame per 4 to 6 s for enhanced phase contrast microscopy and 1 frame per 4 to 30 s for fluorescence microscopy. To estimate the velocity of retrograde flow, time-lapse image sequences were analyzed using kymograph function of Metamorph software. This function copies selected narrow regions from the sequential frames of the time-lapse sequence and pastes them side-by-side in a time-montage. Moving features are visualized in kymographs as diagonal streaks with a slope depending on the velocity of movement.

## Supporting Information

Movie S1 Enhanced phase contrast image sequence of spreading Swiss 3T3 cell (corresponds to Fig. 1A). Field of view, 46 µm×31 µm, images taken at 4 s intervals, total elapsed time, 3 min 20 s.
(0.82 MB MOV)

Movie S2 Enhanced phase contrast and fluorescence microscopy image sequence of spreading REF-52 cell injected with rhodamine-actin (corresponds to Fig. 1B). Field of view, 14 µm×23 µm, images in each mode are taken at 8 s intervals, total elapsed time, 10 min.
(1.09 MB MOV)

Movie S3 Enhanced phase contrast and fluorescence microscopy image sequence of spreading B16 melanoma cell expressing GFP-actin treated with cytochalasin D (corresponds to Fig. 1C). Cytochalasin D-treatment results in the immediate arrest of protrusion and the inhibition of the retrograde flow in the lamellipodium, while the overall actin organization remains virtually unchanged up to 2 min after the addition of the drug. Field of view, 29 µm×44 µm, images in each mode are taken at 10 s intervals, total elapsed time, 8 min 30 s, cytochalasin D is added at 6 min 20 s.
(0.82 MB MPG)

Movie S4 Enhanced phase contrast image sequence of spreading Swiss 3T3 cell treated with rho-kinase inhibitor HA-1077 (corresponds to Fig. 1d). Addition of HA-1077 results in overall spreading of the cell, disappearance of stress fibers in the lamellum and the inhibition of retrograde flow in the lamellum, while the lamellipodium dynamics is not affected. Field of view, 51 µm×38 µm, images are taken at 6 s intervals, total elapsed time, 15 min 30 s, HA-1077 added at 5 min 40 s.
(2.58 MB MOV)

Movie S5 Merged enhanced phase contrast and fluorescence microscopy image sequence of spreading REF-52 cell excpressing YFP-paxillin (corresponds to Fig. 2a). Field of view, 75 µm×55 µm, phase contrast images are taken at 5 s intervals, fluorescenece images, at 20 s intervals, total elapsed time, 6 min.
(1.19 MB MOV)

Movie S6 Zoomed region of merged enhanced phase contrast and fluorescence image sequence of spreading REF-52 cell excpressing YFP-paxillin shows two instances of the formation of new FAs and the associated advance of the lamellum/lamellipodium boundary (corresponds to Fig. 2b). Field of view, 16.3 µm×12.5 µm, phase contrast images are taken at 5 s intervals, fluorescenece images, at 20 s intervals, total elapsed time, 1 min 40 s.
(0.38 MB MOV)

Movie S7 Zoomed region of merged enhanced phase contrast and fluorescence image sequence of spreading REF-52 cell excpressing YFP-paxillin shows an instance of the formation of new FAs and the associated ruffling of the lamellipodium and the advance of the lamellum/lamellipodium boundary (corresponds to Fig. 2c). Field of view, 12.6 µm×8.7 µm, phase contrast images are taken at 5 s intervals, fluorescenece images, at 20 s intervals, total elapsed time, 5 min.
(0.96 MB MOV)

Movie S8 Double fluorescence image sequence of spreading REF-52 cell expressing YFP-paxillin (shown in red) and microinjected with rhodamine-actin (shown in cyan). Numerous new FAs form in the lamellipodium followed by the advance of the lamellipodium/lamellum boundary to the sites of FAs. Field of view, 25 µm×19 µm, total elapsed time, 8 min.
(0.74 MB MOV)

Movie S9 Fluorescence (left) and merged fluorescence and enhanced phase contrast image sequence (right) of spreading REF-52 cell expressing YFP-paxillin (shown in yellow). The moving boundary between the lamellum and the lamellipodium coincides with the row of small FAs in the process of continuous turnover. Field of view, 12.2 µm×16.7 µm, phase contrast images are taken at 6 s intervals, fluorescenece images, at 18 s intervals, total elapsed time, 10 min
(1.30 MB MOV)

Movie S10 Enhanced phase contrast image sequence of REF-52 cell spreading on poly-L-lysin-coated glass surface shows uniform retrograde flow at the cell periphery. Field of view, 41 µm×31 µm, images are taken at 5 s intervals, total elapsed time, 12 min.
(2.34 MB MOV)

Movie S11 Movie S11. Double color fluorescence image sequence of spreading REF-52 cell expressing YFP-paxillin (red) and microinjected with rhodamine-myosin II (cyan) (corresponds to Fig. 4a). Field of view 34 µm×24 µm, images in each channel are taken at 30 s intervals, total elapsed time,12 min.
(0.38 MB MOV)

Movie S12 Double color fluorescence image sequence of REF-52 cell expressing YFP-paxillin (red) and GFP-actin (cyan) (corresponds to Fig. 4b). Field of view 16 µm×4 µm, images in each channel are taken at 20 s intervals, total elapsed time, 8 min.
(0.19 MB MOV)
